# Immunoproteomics to identify species-specific antigens in *Neospora caninum* recognised by infected bovine sera[Fn FN1]

**DOI:** 10.1051/parasite/2022059

**Published:** 2022-12-21

**Authors:** Ruenruetai Udonsom, Onrapak Reamtong, Poom Adisakwattana, Supaluk Popruk, Charoonluk Jirapattharasate, Yoshifumi Nishikawa, Tawin Inpankaew, Jitbanjong Toompong, Manas Kotepui, Aongart Mahittikorn

**Affiliations:** 1 Department of Protozoology, Faculty of Tropical Medicine, Mahidol University Bangkok 10400 Thailand; 2 Department of Molecular Tropical Medicine and Genetics, Faculty of Tropical Medicine, Mahidol University Bangkok 10400 Thailand; 3 Department of Helminthology, Faculty of Tropical Medicine, Mahidol University Bangkok 10400 Thailand; 4 Department of Pre-clinic and Animal Science, Faculty of Veterinary Science, Mahidol University Salaya Nakhon Pathom 73170 Thailand; 5 National Research Center for Protozoan Diseases, Obihiro University of Agriculture and Veterinary Medicine Obihiro Hokkaido 080-8555 Japan; 6 Department of Parasitology, Faculty of Veterinary Medicine, Kasetsart University Bangkok 10900 Thailand; 7 Department of Parasitology, Faculty of Veterinary Medicine, Mahanakorn University of Technology Bangkok 10530 Thailand; 8 Medical Technology, School of Allied Health Sciences, Walailak University Tha Sala Nakhon Si Thammarat 80160 Thailand

**Keywords:** Immunoproteomics, *Neospora caninum*, Mass spectrometry, Bovine host, Apicomplexa

## Abstract

Bovine neosporosis is a disease of concern due to its global distribution and significant economic impact through massive losses in the dairy and meat industries. To date, there is no effective chemotherapeutic drug or vaccine to prevent neosporosis. Control of this disease is therefore dependent on efficient detection tests that may affect treatment management strategies. This study was conducted to identify the specific immunoreactive proteins of *Neospora caninum* tachyzoites recognised by sera from cattle infected with *N. caninum*, *Toxoplasma gondii*, *Cryptosporidium parvum*, *Babesia bovis* and *B. bigemina*, and by sera from uninfected cattle using two-DE dimensional gel electrophoresis (2-DE) combined with immunoblot and mass spectrometry (LC-MS/MS). Among 70 protein spots that reacted with all infected sera, 20 specific antigenic spots corresponding to 14 different antigenic proteins were recognised by *N. caninum*-positive sera. Of these immunoreactive antigens, proteins involved in cell proliferation and invasion process were highly immunogenic, including HSP90-like protein, putative microneme 4 (MIC4), actin, elongation factor 1-alpha and armadillo/beta-catenin-like repeat-containing protein. Interestingly, we discovered an unnamed protein product, rhoptry protein (ROP1), possessing strong immunoreactivity against *N. caninum* but with no data on function available. Moreover, we identified cross-reactive antigens among these apicomplexan parasites, especially *N. caninum*, *T. gondii* and *C. parvum*. *Neospora caninum*-specific immunodominant proteins were identified for immunodiagnosis and vaccine development. The cross-reactive antigens could be evaluated as potential common vaccine candidates or drug targets to control the diseases caused by these apicomplexan protozoan parasites.

## Abstract (fr)

La néosporose bovine est une maladie préoccupante en raison de sa distribution mondiale et de son impact économique important par d’énormes pertes dans les industries laitières et de la viande. À ce jour, il n’existe aucun médicament chimiothérapeutique ou vaccin efficace pour prévenir la néosporose. Par conséquent, le contrôle de cette maladie dépend de tests de détection efficaces qui affecteraient les stratégies de gestion du traitement. Cette étude a été menée pour identifier les protéines immunoréactives spécifiques des tachyzoïtes de *Neospora caninum* reconnues par les sérums de bovins infectés par *N. caninum*, *Toxoplasma gondii*, *Cryptosporidium parvum*, *Babesia bovis* et *B. bigemina* et par les sérums de bovins non infectés, à l’aide d’un gel d’électrophorèse bidimensionnel (2DE) combiné à l’immunoblot et à la spectrométrie de masse (LC-MS/MS). Parmi 70 spots protéiques ayant réagi avec tous les sérums infectés, 20 spots antigéniques spécifiques correspondant à 14 protéines antigéniques différentes ont été reconnus par les sérums positifs à *N. caninum*. Parmi ces antigènes immunoréactifs, les protéines impliquées dans la prolifération cellulaire et le processus d’invasion étaient hautement immunogènes, notamment la protéine de type HSP90, le micronème putatif 4 (MIC4), l’actine, le facteur d’élongation 1-alpha et la protéine à répétition de type armadillo/bêta-caténine. Fait intéressant, nous avons découvert un produit protéique sans nom, la protéine de rhoptries (ROP1), possédant une forte immunoréactivité contre *N. caninum* mais sans données disponibles sur sa fonction. De plus, nous avons identifié des antigènes à réaction croisée parmi ces parasites apicomplexes, en particulier *N. caninum*, *T. gondii* et *C. parvum*. Des protéines immunodominantes spécifiques de *Neospora caninum* ont été identifiées pour l’immunodiagnostic et le développement de vaccins. Les antigènes à réaction croisée pourraient être évalués comme candidats vaccins communs potentiels ou comme cibles médicamenteuses pour contrôler les maladies causées par ces parasites protozoaires apicomplexes.

## Introduction

*Neospora caninum* is an apicomplexan protozoan parasite and a primary cause of abortion in cattle throughout the world [16]. Consequently, bovine neosporosis is currently a disease of concern worldwide due to its global distribution and significant economic impact through massive losses in the dairy and meat industries [15, 20, 37]. Currently, no effective drugs or vaccines are available to prevent abortion or transmission caused by *N. caninum* infection in cattle [20]. *Neospora caninum* infection is generally latent and asymptomatic in non-pregnant cattle, although persistent infection throughout life is an important feature of bovine neosporosis, resulting in repeated abortions by transplacental (vertical) transmission, the principal route of infection [4, 8]. Prevention and control strategies of neosporosis are dependent on farm management practices and strict hygiene. At present, serological diagnosis is the only option to discriminate between infected and uninfected animals, followed by appropriate treatment management to control bovine neosporosis [19]. Several diagnostic methods for bovine neosporosis are used to detect specific antibodies against *N. caninum*. The enzyme-linked immunosorbent assay (ELISA) and indirect immunofluorescence antibody test (IFAT) are the most common techniques used to diagnose *N. caninum* infections [2, 6]. However, a major problem concerning conventional serological testing is the possibility of low specificity of diagnosis due to cross-reactivity among closely related apicomplexan pathogens, including *Toxoplasma gondii*, *Cryptosporidium parvum* and *Babesia* spp. (*B. bovis* and *B. bigemina*) [18].

There are reports of serological cross-reactivity among animals infected with *N. caninum* and *T. gondii*. Cross-reactive *N. caninum* soluble antigens (NLA) were recognised using sera from mice and cats immunised with *T. gondii* [33]. Of 384 monoclonal antibodies (mAbs), 10 were produced by immunising mice with *N. caninum* tachyzoites and were found to be cross-reactive between *N. caninum* and *T. gondii*. Among these, three antigenic proteins, including protein disulfide isomerase (PDI), heat shock protein 70 (HSP70) and ribosomal protein 1 (RP1), were identified as cross-reactive antigens between both parasites [28]. Similarly, Sohn *et al*. developed a panel of 46 mAbs using a mouse immunised with a mixed fraction of *N. caninum* organelles and found that some of these mAbs cross-reacted with *T. gondii* [41]. Current investigations of the parasite proteome provide comprehensive insights into their biological processes and highlight valuable diagnostic biomarkers, as well as new vaccine targets [44]. It is necessary to identify parasite-specific proteins to develop novel and specific biomarkers to enhance sensitivity and specificity for precise and acceptable diagnosis. The initial proteomics analysis of *N. caninum* tachyzoite conducted by Lee *et al*. revealed 31 spots corresponding to 20 different proteins identified from *N. caninum* tachyzoites by peptide mass fingerprinting and 17 spots corresponding to 11 antigenic proteins identified from *N. caninum* protein map [27]. Another study identified 64 spots as antigenic proteins on immunoblot profiles using rabbit anti-sera [25]. A comparison of proteomes between *N. caninum* and *T. gondii* tachyzoites was also conducted, which revealed the cross-reactive antigens between them [26, 48]. Currently, there are limited proteomics studies on the species-specific antigens or cross-reactivity of *N. caninum* compared with other apicomplexan parasites in the bovine host. Therefore, this study was conducted to identify the immunoreactive and antigenic proteins of *N. caninum* tachyzoites using infected bovine sera specific to *N. caninum*, *T. gondii*, *C. parvum*, *B. bovis* and *B. bigemina* and healthy host sera by immunoproteomics. MS and bioinformatics analyses were performed to identify and characterise the cross-reactive and species-specific antigens among these parasites. These species-specific immunogenic proteins could be targeted as new biomarkers for *N. caninum* immunodiagnosis or vaccine development.

## Materials and methods

### Bovine immune serum samples

Fourteen bovine serum samples infected with the five protozoan parasites *N. caninum* (*N* = 2), *T. gondii* (*N* = 3), *C. parvum* (*N* = 3), *B. bovis* (*N* = 4) and *B. bigemina* (*N* = 2) and negative sera (*N* = 4) were used in this study. The details of bovine serum samples are presented in Table 1. This study was approved by the Faculty of Tropical Medicine-Animal Care and Use Committee, Mahidol University (FTM-ACUC 005/2022E).

**Table 1:** List of known bovine serum samples used this study.

Protozoan-infection	No. sample (*N* = 18)	Confirmed tests	Background of sample	Source
*N. caninum*	2	IFAT, iELISA against NcSAG1	Male Holstein calves inoculated intravenously with *N. caninum* (Nc-1) tachyzoites	[1]
*T. gondii*	3	IFAT, PCR and IHC	Pregnant heifers inoculated intravenously with *T. gondii* (RH) tachyzoites	[47]
*C. parvum*	3	Commercial-ICT kit (Bio-X Diagnostics SPRL, Jemelle, Belgium), Faecal examination	Calves had experienced cryptosporidiosis	[21]
*B. bovis*	4	iELISA, ICT against SBP4	Field specimen	Faculty of Veterinary Medicine, Kasetsart University, Thailand
*B. bigemina*	2	iELISA and ICT against RAP1	Field specimen	Faculty of Veterinary Medicine, Kasetsart University, Thailand
Healthy bovine serum	4	IFAT, iELISA	Male Holstein calves prior parasites inoculation	[1]

IFAT, indirect fluorescent antibody test; IHC, immunohistochemistry; iELISA, indirect enzyme-linked immunosorbent assay; ICT, immunochromatographic test; PCR, polymerase chain reaction.

### Maintenance and purification of *N. caninum*

*Neospora caninum* tachyzoites (Nc-1 strain) were maintained in African green monkey kidney (Vero) cell monolayer with Dulbecco’s Modified Eagle Medium (Cytiva HyClone™) supplemented with 10% foetal bovine serum, L-glutamine (2 mM/mL), penicillin–streptomycin (100 U/mL penicillin and 100 μg/mL streptomycin) and amphotericin B (0.25 μg/mL) in a humidified atmosphere with 5% CO_2_ at 37 °C. *Neospora caninum* tachyzoites were harvested by cell scraping of the infected Vero host cell after 3–4 days of inoculation. The tachyzoites were separated from host cells using a 5 μm filter, and the tachyzoite suspension was loaded onto a 30%, 50% and 80% (v/v) osmotic Percoll^®^ (Sigma-Aldrich, Burlington, MA, USA) gradient to purify and eliminate the remaining host cells, followed by centrifugation at 2,000 *g* for 30 min at 4 °C. The viable tachyzoite band forming between the 50% and 80% osmotic Percoll^®^ gradients were collected and washed three times with phosphate-buffered saline (PBS). The purified *N. caninum* tachyzoite pellet was stored at −70 °C until use [27].

### Preparation of soluble *N. caninum* proteins

Purified *N. caninum* tachyzoites were dissolved in a lysis buffer containing 8 M urea, 2 M thiourea, 4% (w/v) CHAPS and 50 mM DTT, and then the *N. caninum* sample was sonicated at 5.5 W for 2 min (5 s pulse/10 s rest) on ice slurry. The suspension was centrifuged at 14,000 rpm for 30 min at 4 °C, and the resulting supernatant was collected. Protein concentration was estimated by the Bradford assay (BioRad Inc., Hercules, CA, USA) using bovine serum albumin as a standard, and the interfering substance was removed using a 2-D Clean-Up Kit (GE Healthcare Bioscience, Chalfont St Giles, UK), according to the manufacturer’s protocol.

### Two-dimensional electrophoresis

Immobiline^TM^ DryStrip gels (pH 3–10, NL 7 cm; GE Healthcare, Uppsala, Sweden) were rehydrated overnight at 25 °C with *N. caninum* soluble protein (100 μg/strip), 1% (w/v) bromophenol blue and 0.5% IPG buffer (pH 3–10, NL; GE Healthcare). Isoelectric focusing (IEF) was performed using an Ettan IPG Phor Electrofocusing system (GE Healthcare) under the following running conditions: 0.2 kV/h for the initial 30 min, followed by a gradient of 0.3 kV/h for 30 min, 4.5 kV/h for 90 min and step down and hold at 3.0 kV/h for 35 min. After IEF, the IPG strips were equilibrated in 5 mg/mL dithiothreitol (DTT) for 15 min and 25 mg/mL iodoacetamide for 15 min, and each focused IPG strip was inserted into 12% sodium dodecyl sulphate polyacrylamide gel and sealed with 0.5% agarose gel. Electrophoresis was conducted at 150 V per gel until the bromophenol blue dye reached the lower gel edge. Protein spots were visualised by silver staining, and the immunoreactive spots in these gels (three gels) were excised and pooled for mass spectrometric analysis. Other six gels from two-dimensional gel electrophoresis (2-DE) were used for immunomics analysis.

### Immunomics analysis

For immunoblotting studies, the proteins on the 2-DE gels were transblotted onto nitrocellulose membranes (Merck Millipore, Carrigtwohill, Ireland) using the western blot wet/tank transfer (Amersham Bioscience, Amersham, UK) under the running conditions of 20 V, 400 mA at 4 °C, overnight. The blotted membranes were blocked with 5% skimmed milk in PBS containing 0.05% Tween-20 (PBS-T) for 1 h and then probed with pooled bovine sera (diluted 1:400) confirmed to be infected with apicomplexan parasites, including *N. caninum* (*N* = 2), *T. gondii* (*N* = 3), *C. parvum* (*N* = 3), *B. bovis* (*N* = 4) and *B. bigemina* (*N* = 2) for 2 h. Pooled healthy bovine sera (*N* = 4) with no history of infection were used as negative controls. After washing with PBS-T, the membranes were incubated with horseradish peroxidase (HRP)-conjugated anti-bovine immunoglobulin G (Invitrogen, Waltham, MA, USA) at 1:2,000 dilution for 1 h. Ultra TMB-Blotting solution substrate (Thermo Fisher Scientific, Milton Park, UK) was used to visualise the antigen–antibody reactive spots, and then the protein spots specific to *N. caninum* infection were identified and compared with the immunoreactive spots specific to other apicomplexan parasites. Immunoreactive protein spots were excised from the silver-stained 2-DE gels and subjected to trypsin digestion.

### In-gel digestion

Immunoreactive protein spots were manually excised from the silver-stained 2-DE gels. Gel pieces were de-stained at 4 °C overnight with 50% acetonitrile (ACN; Sigma-Aldrich) in 50 mM ammonium bicarbonate (Merck). The disulfide bonds in the proteins were reduced with 4 mM DTT in 50 mM ammonium bicarbonate at 60 °C for 15 min and then alkylated with 250 mM iodoacetamide at room temperature for 30 min in the dark. The reaction was quenched by 4 mM DTT in 50 mM ammonium bicarbonate for 5 min at room temperature, after which the entire solution was removed, and the gel pieces were dehydrated with acetonitrile. The gel pieces were digested with proteomics-grade trypsin (Sigma-Aldrich) in 50 mM ammonium bicarbonate at 37 °C overnight. The digested peptides were extracted by acetonitrile and dried in a vacuum centrifuge.

### Mass spectrometry analysis (LC-MS/MS)

Dried tryptic peptides were redissolved in 0.1% formic acid. Each sample was injected and analysed for amino acid sequences using the UltiMate 3000 nano-liquid chromatography (nano-LC) system (Dionex, Camberley, UK). The mass spectra obtained from the mass spectrometry (MS) and tandem mass spectrometry (MS/MS) covered mass ranges of m/z 400–2000 and m/z 50–1500, respectively. A mascot generic file (.mgf) was generated using the data analysis software (Bruker Daltonics, Billerica, MA, USA). Mascot Daemon version 2.3.2 (Matrix Science, London, UK) was used to merge the .mgf files and identified the proteins. The National Center for Biotechnology Information (NCBI) (https://www.ncbi.nlm.nih.gov/) was set as the protein sequence database, and ToxoDB *Toxoplasma* informatics resources (https://toxodb.org/toxo/app) were also used for protein identification. Peptides with 95% confidence were reported in this study to reduce false-positive results.

## Results

### 2-DE profile of *N. caninum* tachyzoite proteins

**Figure 1:**
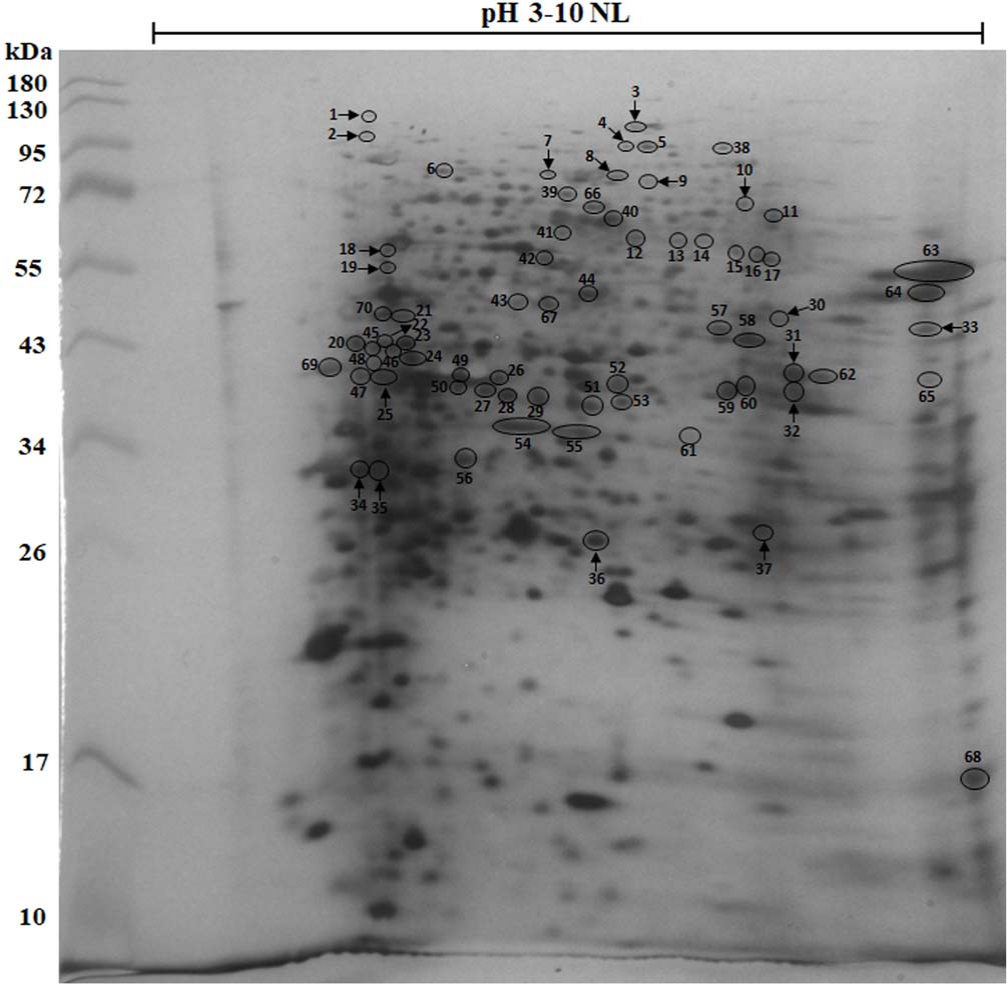
Two-dimensional electrophoresis protein patterns of *N. caninum* (Nc-1) tachyzoites separated using 12% acrylamide and visualised by silver staining. A total of 70 immunoreactive protein spots (spot numbers 1–70) were identified on the 2-DE gel based on immunoblot analysis that were recognised by known bovine sera. The circle or ellipse with arrows indicates 20 specific protein spots that were recognised by *N. caninum*-positive bovine pooled sera.

The 2-DE analysis followed by silver staining revealed approximately 500–600 protein spots, and most protein spots were located between 10 and 130 kDa (Fig. 1). Based on 2-DE immunoblotting, 70 immunoreactive spots were identified that are indicated using circles or ellipse in the figure. Among these, 37 spots (spot numbers 1–37) were recognised by anti-*N. caninum* serum, and only 20 protein spots marked with arrows (spot numbers 1, 2, 3, 4, 7, 8, 9, 10, 18, 19, 22, 25, 30, 31, 32, 33, 34, 35, 36 and 37) corresponding to 14 different antigenic proteins were specific to *N. caninum*. Approximately 50 protein spots were cross-reactive with other apicomplexan-infected sera. The antigenic spots were most abundant at molecular masses ranging from 26 to 130 kDa. All the immunoreactive protein spots were excised and identified by LC-MS/MS. Protein identification was performed by MASCOT search engine 2.3 (Matrix Science, Ltd.) using the NCBI *N. caninum* database. Table 2 shows the data of these spots consisting of identification scores, molecular weight, number of matched peptides, percentage of protein sequence coverage and isoelectric point (p*I*).

**Table 2:** List of proteins identified on the 2-DE profiles of *Neospora caninum* (Nc-1) tachyzoites probed with known bovine sera analysed by mass spectrometry (LC-MS/MS).

Spot	Accession No.	Protein name	Score^a^	M.W.^b^	No. of peptide^c^	% coverage^d^	p*I* *e*
1	CBZ52122.1	HSP90-like protein, related [*N. caninum* Liverpool]	89	96,996	3	6.5	4.91
2	CBZ53664.1	Hypothetical protein NCLIV_034460 [*N. caninum* Liverpool]	281	64,657	8	18.9	4.82
3	CEL75932.1	Ubiquitin carboxyl-terminal hydrolase, putative [*T. gondii* VEG]	42	247,984	2	1.1	5.75
4	CEL76857.1	corA-like Mg2 transporter domain-containing protein [*T. gondii* VEG]	53	126,373	1	0.8	9
5	CBZ52236.1	Putative elongation factor 2 [*N. caninum* Liverpool]	131	92,975	5	10.1	5.93
6	CBZ56259.1	Hypothetical protein NCLIV_066840 [*N. caninum* Liverpool]	421	79,021	10	21.2	7.48
7	CEL76857.1	corA-like Mg2 transporter domain-containing protein [*T. gondii* VEG]	54	126,373	2	2.6	9
8	CBZ49807.1	Putative microneme protein MIC4 [*N. caninum* Liverpool]	205	64,427	7	17.8	6.29
9	CEL76857.1	corA-like Mg2 transporter domain-containing protein [*T. gondii* VEG]	57	126,373	2	2.6	9
10	CEL67492.1	Inosine-5′-monophosphate dehydrogenase [*N. caninum* Liverpool]	395	61,250	13	31	7.25
11	CBZ53094.1	Glucose-6-phosphate isomerase, related [*N. caninum* Liverpool]	122	53,628	3	8.5	6.73
12	CBZ50563.1	Putative dihydrolipoamide branched chain transacylase, E2 subunit [*N. caninum* Liverpool]	423	69,400	10	18.4	6.2
13	CBZ55235.1	Putative phosphatidylinositol-4-phosphate 5-kinase [*N. caninum* Liverpool]	44	50,948	1	5.2	6.93
14	CBZ54369.1	Conserved hypothetical protein [*N. caninum* Liverpool]	49	924,393	5	0.9	6.44
15	CBZ52433.1	Hypothetical protein NCLIV_022220 [*N. caninum* Liverpool]	211	63,160	7	15.5	8.56
16	CEL65811.1	Putative 2-hydroxyacid dehydrogenase SACOL2296 [*N. caninum* Liverpool]	181	65,408	5	13.7	6.46
17	CBZ51198.1	gg11844, related [*N. caninum* Liverpool]	270	49,423	7	20	7.38
18	CBZ49859.1	Actin, related [*N. caninum* Liverpool]	347	41,881	10	28.5	5.05
19	CBZ49859.1	Actin, related [*N. caninum* Liverpool]	111	41,881	4	15.4	5.05
20	CEL69162.1	Ubiquitin, putative [*N. caninum* Liverpool]	154	40,879	4	18.3	4.79
21	AAC15250.1	Surface protein Nc-p43 [*N. caninum*]	81	42,025	2	7.8	5.49
22	CEL76857.1	corA-like Mg2 transporter domain-containing protein [*T.* gondii VEG]	53	126,373	1	0.8	9
23	CBZ50660.1	Hypothetical protein NCLIV_011270 [*N. caninum* Liverpool]	530	44,623	15	41.6	5.61
24	CBZ54372.1	Hypothetical protein NCLIV_048020 [*N. caninum* Liverpool]	288	36,035	7	27.1	5.63
25	CEL76857.1	corA-like Mg2 transporter domain-containing protein [*T. gondii* VEG]	52	126,373	1	0.8	9
26	CEL76857.1	corA-like Mg2 transporter domain-containing protein [*T. gondii* VEG]	53	126,373	1	0.8	9
27	CBZ56165.1	Conserved hypothetical protein [*N. caninum* Liverpool]	468	33,610	9	46.9	5.6
28	CBZ55758.1	60S acidic ribosomal protein P0 [*N. caninum* Liverpool]	507	33,843	11	51.4	5.44
29	CBZ54609.1	Unnamed protein product [*N. caninum* Liverpool]	223	38,998	10	33.1	7.01
30	CBZ49956.1	Hypothetical protein NCLIV_004400 [*N. caninum* Liverpool]	239	50,651	6	18.7	8.51
31	CBZ54957.1	Unnamed protein product [*N. caninum* Liverpool]	115	38,797	2	8.2	5.82
32	CBZ54957.1	Unnamed protein product [*N. caninum* Liverpool]	156	38,797	3	11.5	5.82
33	CBZ49679.1	Elongation factor 1-alpha, related [*N. caninum* Liverpool]	66	49,018	2	4	9.02
34	CBZ50039.1	Hypothetical protein NCLIV_005150 [*N. caninum* Liverpool]	177	50,033	4	12.7	4.7
35	CBZ52818.1	Armadillo/beta-catenin-like repeat-containing protein [*N. caninum* Liverpool]	60	30,504	3	15	5.01
36	CBZ54937.1	Putative peroxidoxin 2 [*N. caninum* Liverpool]	58	24,518	2	8.9	5.84
37	CBZ52736.1	Putative Gbp1p protein [*N. caninum* Liverpool]	80	31,836	3	15.3	9.15
38	CBZ54173.1	Hypothetical protein NCLIV_046050 [*N. caninum* Liverpool]	45	131,192	2	2.8	6.05
39	CBZ52239.1	Hypothetical protein NCLIV_020250 [*N. caninum* Liverpool]	149	60,778	4	7.9	5.83
40	CBZ51726.1	Pyruvate kinase, related [*N. caninum* Liverpool]	474	57,581	10	28.8	6.01
41	CEL76893.1	Dynein 1-beta heavy chain, flagellar inner arm I1 complex, putative [*T. gondii* VEG]	79	518,185	7	2.5	6.05
42	CEL76893.1	Dynein 1-beta heavy chain, flagellar inner arm I1 complex, putative [*T. gondii* VEG]	37	518,185	2	0.5	6.05
43	CBZ54957.1	Unnamed protein product [*N. caninum* Liverpool]	203	38,797	5	14.6	5.82
44	CEL66738.1	Elongation factor Tu, putative [*N. caninum* Liverpool]	156	59,096	7	17.6	8.23
45	CEL71761.1	Hypothetical protein BN1205_038970 [*T. gondii* VEG]	57	50,170	1	3.3	5.15
46	CBZ50660.1	Hypothetical protein NCLIV_011270 [*N. caninum* Liverpool]	202	44,623	5	17.2	5.61
47	CBZ53299.1	Conserved hypothetical protein [*N. caninum* Liverpool]	63	61,681	3	9.9	4.78
48	CEL76857.1	corA-like Mg2 transporter domain-containing protein [*T. gondii* VEG]	45	126,373	1	0.8	9
49	CBZ53399.1	Putative serine-threonine phosphatase 2C [*N. caninum* Liverpool]	261	35,552	7	29	5.35
50	CBZ53399.1	Putative serine-threonine phosphatase 2C [*N. caninum* Liverpool]	120	35,552	4	18.1	5.35
51	CEL76857.1	corA-like Mg2 transporter domain-containing protein [*T. gondii* VEG]	53	126,373	1	0.8	9
52	CEL76857.1	corA-like Mg2 transporter domain-containing protein [*T. gondii* VEG]	53	126,373	1	0.8	9
53	CEL76857.1	corA-like Mg2 transporter domain-containing protein [*T. gondii* VEG]	46	126,373	2	2.6	9
54	CAA06661.1	p36 protein [*N. caninum*]	457	33,049	11	37.3	7.89
55	CAA06661.1	p36 protein [*N. caninum*]	136	33,049	4	19.1	7.89
56	CBZ51110.1	Putative Hsp20/alpha crystalline domain-containing protein [*N. caninum* Liverpool]	210	29,682	7	29.5	5.56
57	CBZ53437.1	Catalase (EC 1.11.1.6), related [*N. caninum* Liverpool]	129	58,700	5	13.6	6.75
58	CBZ54609.1	Unnamed protein product [*N. caninum* Liverpool]	1235	38,998	24	59.1	7.01
59	CBZ51119.1	Glyceraldehyde 3-phosphate dehydrogenase, related [*N. caninum* Liverpool]	425	36,452	9	39.7	6.83
60	CBZ51119.1	glyceraldehyde 3-phosphate dehydrogenase, related [*N. caninum* Liverpool]	518	36,452	11	40.3	6.83
61	CEL68024.1	Hypothetical protein BN1204_038021 [*N. caninum* Liverpool]	49	191,022	4	2.4	5.03
62	CBZ49679.1	Elongation factor 1-alpha, related [*N. caninum* Liverpool]	59	49,018	1	2.5	9.02
63	CBZ49679.1	Elongation factor 1-alpha, related [*N. caninum* Liverpool]	866	49,018	21	52	9.02
64	CBZ49679.1	Elongation factor 1-alpha, related [*N. caninum* Liverpool]	655	49,018	17	43.8	9.02
65	CEL78445.1	ATP-dependent DNA helicase II, 70 kDa subunit, putative [*T. gondii* VEG]	39	94,541	2	3.1	4.95
66	CEL78039.1	Pantothenate kinase, putative [*T. gondii* VEG]	59	125,264	4	6.9	9.06
67	CEL71084.1	Protein phosphatase 2C, putative [*N. caninum* Liverpool]	123	46,413	3	9.2	5.94
68	CBZ52637.1	Hypothetical protein NCLIV_024250 [*N. caninum* Liverpool]	162	16,436	4	17.7	10.4
69	CBZ53255.1	Rcn2-prov protein, related [*N. caninum* Liverpool]	227	39,265	6	20	4.65
70	CBZ53608.1	Heat shock protein 70, related [*N. caninum* Liverpool]	189	72,613	4	5.4	5.07

a Identification score.

b Molecular weight.

c Number of matched peptides.

d Percentage of protein sequence covered by matched peptides.

e Isoelectric point.

### Detection of immunoreactive spots by 2-DE immunoblotting using immune bovine sera against *N. caninum* and other apicomplexan parasites

**Figure 2:**
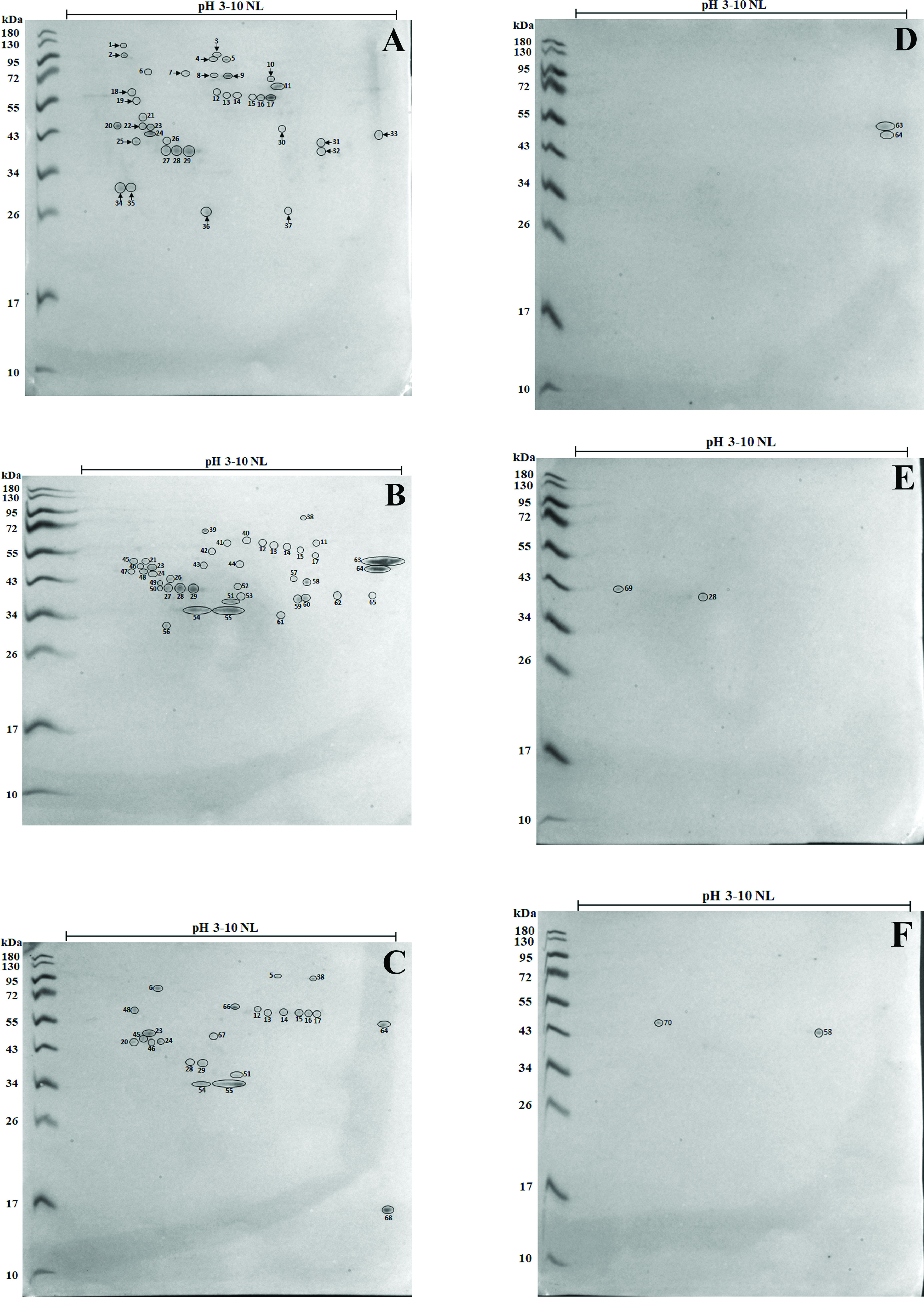
Immunoblot analysis of 2-DE-separated *N. caninum* tachyzoite antigens using pooled bovine anti-*N. caninum* (A), anti-*T. gondii* (B), anti-*C. parvum* (C), anti-*B. bovis* (D) and anti-*B. bigemina* (E) sera and healthy serum (F). The arrows indicate 20 protein spots corresponding to 14 different antigenic proteins recognised by *N. caninum*-positive sera, and those without arrows indicate spots cross-reactive with other apicomplexan protozoan-infected sera.

The immunoreactive protein spots recognised in individual bovine sera on the 2-DE immunoblot profiles are shown in Figures 2A–2F. A total of 37 immunoreactive protein spots were recognised by anti-*N. caninum* serum, but only 20 antigenic protein spots (indicated with arrows) corresponding to 14 different proteins were specific to *N. caninum* (Fig. 2A). The immunoblot analysis also revealed 41 protein spots recognised by anti-*T. gondii* serum (Fig. 2B), 24 by anti-*C. parvum* serum (Fig. 2C) and 2 by anti-*B. bovis* and anti*-B. bigemina* sera (Fig. 2D and 2E). Furthermore, two protein spots were detected by healthy bovine sera (Fig. 2F). At least 50 protein spots were identified to exhibit cross-reactivity of *N. caninum* tachyzoite proteins with other apicomplexan parasites. Most protein spots were cross-reactive with the closely related *T. gondii* and *C. parvum*. Spot numbers 12, 13, 14, 15, 17, 23, 24 and 29 were recognised by anti-*N. caninum* sera but demonstrated cross-reactivity with anti-*T. gondii* and anti-*C. parvum* sera. Spot numbers 38, 45, 46, 48, 51, 54 and 55 were also cross-reactive against anti-*T. gondii* and anti-*C. parvum* sera. Spot number 64 was cross-reactive against anti-*T. gondii*, anti-*C. parvum* and anti-*B. bovis* sera, whereas spot number 28 showed cross-reactivity with anti-*T. gondii*, anti-*C. parvum* and anti-*B. bigemina* sera. All immunogens were classified according to their specific reactivity against *N. caninum* and other apicomplexan parasite infections in bovine hosts and are listed in Table 3.

**Table 3:** List of proteins identified as specific and/or cross-reactive antigens of *Neospora caninum* and other apicomplexan protozoa in the bovine host.

Spot	Accession No.	Protein name	Anti-*N. caninum* serum	Anti-*T. gondii* serum	Anti-*C. parvum* serum	Anti-*B. bovis* serum	Anti-*B. bigemina* serum	Healthy serum
1	CBZ52122.1	HSP90-like protein, related [*N. caninum* Liverpool]	+^a^	−	−	−	−	−
2	CBZ53664.1	Hypothetical protein NCLIV_034460 [*N. caninum* Liverpool]	+^a^	−	−	−	−	−
3	CEL75932.1	Ubiquitin carboxyl-terminal hydrolase, putative [*T. gondii* VEG]	+^a^	−	−	−	−	−
4	CEL76857.1	corA-like Mg2 transporter domain-containing protein [*T. gondii* VEG]	+^a^	−	−	−	−	−
5	CBZ52236.1	Putative elongation factor 2 [*N. caninum* Liverpool]	+	−	+	−	−	−
6	CBZ56259.1	Hypothetical protein NCLIV_066840 [*N. caninum* Liverpool]	+	−	+	−	−	−
7	CEL76857.1	corA-like Mg2 transporter domain-containing protein [*T. gondii* VEG]	+^a^	−	−	−	−	−
8	CBZ49807.1	Putative microneme protein MIC4 [*N. caninum* Liverpool]	+^a^	−	−	−	−	−
9	CEL76857.1	corA-like Mg2 transporter domain-containing protein [*T. gondii* VEG]	+^a^	−	−	−	−	−
10	CEL67492.1	Inosine-5′-monophosphate dehydrogenase [*N. caninum* Liverpool]	+^a^	−	−	−	−	−
11	CBZ53094.1	Glucose-6-phosphate isomerase, related [*N. caninum* Liverpool]	+	+	−	−	−	−
12	CBZ50563.1	Putative dihydrolipoamide branched chain transacylase, E2 subunit [*N. caninum* Liverpool]	+	+	+	−	−	−
13	CBZ55235.1	Putative phosphatidylinositol-4-phosphate 5-kinase [*N. caninum* Liverpool]	+	+	+	−	−	−
14	CBZ54369.1	Conserved hypothetical protein [*N. caninum* Liverpool]	+	+	+	−	−	−
15	CBZ52433.1	Hypothetical protein NCLIV_022220 [*N. caninum* Liverpool]	+	+	+	−	−	−
16	CEL65811.1	Putative 2-hydroxyacid dehydrogenase SACOL2296 [*N. caninum* Liverpool]	+	−	+	−	−	−
17	CBZ51198.1	gg11844, related [*N. caninum* Liverpool]	+	+	+	−	−	−
18	CBZ49859.1	Actin, related [*N. caninum* Liverpool]	+^a^	−	−	−	−	−
19	CBZ49859.1	Actin, related [*N. caninum* Liverpool]	+^a^	−	−	−	−	−
20	CEL69162.1	Ubiquitin, putative [*N. caninum* Liverpool]	+	−	+	−	−	−
21	AAC15250.1	Surface protein Nc-p43 [*N. caninum*]	+	+	−	−	−	−
22	CEL76857.1	corA-like Mg2 transporter domain-containing protein [*T. gondii* VEG]	+^a^	−	−	−	−	−
23	CBZ50660.1	Hypothetical protein NCLIV_011270 [*N. caninum* Liverpool]	+	+	+	−	−	−
24	CBZ54372.1	Hypothetical protein NCLIV_048020 [*N. caninum* Liverpool]	+	+	+	−	−	−
25	CEL76857.1	corA-like Mg2 transporter domain-containing protein [*T. gondii* VEG]	+^a^	−	−	−	−	−
26	CEL76857.1	corA-like Mg2 transporter domain-containing protein [*T. gondii* VEG]	+	+	−	−	−	−
27	CBZ56165.1	Conserved hypothetical protein [*N. caninum* Liverpool]	+	+	−	−	−	−
28	CBZ55758.1	60S acidic ribosomal protein P0 [*N. caninum* Liverpool]	+	+	+	−	+	−
29	CBZ54609.1	Unnamed protein product [*N. caninum* Liverpool]	+	+	+	−	−	−
30	CBZ49956.1	Hypothetical protein NCLIV_004400 [*N. caninum* Liverpool]	+^a^	−	−	−	−	−
31	CBZ54957.1	Unnamed protein product [*N. caninum* Liverpool]	+^a^	−	−	−	−	−
32	CBZ54957.1	Unnamed protein product [*N. caninum* Liverpool]	+^a^	−	−	−	−	−
33	CBZ49679.1	Elongation factor 1-alpha, related [*N. caninum* Liverpool]	+^a^	−	−	−	−	−
34	CBZ50039.1	Hypothetical protein NCLIV_005150 [*N. caninum* Liverpool]	+^a^	−	−	−	−	−
35	CBZ52818.1	Armadillo/beta-catenin-like repeat-containing protein [*N. caninum* Liverpool]	+^a^	−	−	−	−	−
36	CBZ54937.1	Putative peroxidoxin 2 [*N. caninum* Liverpool]	+^a^	−	−	−	−	−
37	CBZ52736.1	Putative Gbp1p protein [*N. caninum* Liverpool]	+^a^	−	−	−	−	−
38	CBZ54173.1	Hypothetical protein NCLIV_046050 [*N. caninum* Liverpool]	−	+	+	−	−	−
39	CBZ52239.1	Hypothetical protein NCLIV_020250 [*N. caninum* Liverpool]	−	+	−	−	−	−
40	CBZ51726.1	Pyruvate kinase, related [*N. caninum* Liverpool]	−	+	−	−	−	−
41	CEL76893.1	Dynein 1-beta heavy chain, flagellar inner arm I1 complex, putative [*T. gondii* VEG]	−	+	−	−	−	−
42	CEL76893.1	Dynein 1-beta heavy chain, flagellar inner arm I1 complex, putative [*T. gondii* VEG]	−	+	−	−	−	−
43	CBZ54957.1	Unnamed protein product [*N. caninum* Liverpool]	−	+	−	−	−	−
44	CEL66738.1	Elongation factor Tu, putative [*N. caninum* Liverpool]	−	+	−	−	−	−
45	CEL71761.1	Hypothetical protein BN1205_038970 [*T. gondii* VEG]	−	+	+	−	−	−
46	CBZ50660.1	Hypothetical protein NCLIV_011270 [*N. caninum* Liverpool]	−	+	+	−	−	−
47	CBZ53299.1	Conserved hypothetical protein [*N. caninum* Liverpool]	−	+	−	−	−	−
48	CEL76857.1	corA-like Mg2 transporter domain-containing protein [*T. gondii* VEG]	−	+	+	−	−	−
49	CBZ53399.1	Putative serine-threonine phosphatase 2C [*N. caninum* Liverpool]	−	+	−	−	−	−
50	CBZ53399.1	Putative serine-threonine phosphatase 2C [*N. caninum* Liverpool]	−	+	−	−	−	−
51	CEL76857.1	corA-like Mg2 transporter domain-containing protein [*T. gondii* VEG]	−	+	+	−	−	−
52	CEL76857.1	corA-like Mg2 transporter domain-containing protein [*T. gondii* VEG]	−	+	−	−	−	−
53	CEL76857.1	corA-like Mg2 transporter domain-containing protein [*T. gondii* VEG]	−	+	−	−	−	−
54	CAA06661.1	p36 protein [*N. caninum*]	−	+	+	−	−	−
55	CAA06661.1	p36 protein [*N. caninum*]	−	+	+	−	−	−
56	CBZ51110.1	Putative Hsp20/alpha crystalline domain-containing protein [*N. caninum* Liverpool]	−	+	−	−	−	−
57	CBZ53437.1	Catalase (EC 1.11.1.6), related [*N. caninum* Liverpool]	−	+	−	−	−	−
58	CBZ54609.1	Unnamed protein product [*N. caninum* Liverpool]	−	+	−	−	−	+
59	CBZ51119.1	Glyceraldehyde 3-phosphate dehydrogenase, related [*N. caninum* Liverpool]	−	+	−	−	−	−
60	CBZ51119.1	Glyceraldehyde 3-phosphate dehydrogenase, related [*N. caninum* Liverpool]	−	+	−	−	−	−
61	CEL68024.1	Hypothetical protein BN1204_038021 [*N. caninum* Liverpool]	−	+	−	−	−	−
62	CBZ49679.1	Elongation factor 1-alpha, related [*N. caninum* Liverpool]	−	+	−	−	−	−
63	CBZ49679.1	Elongation factor 1-alpha, related [*N. caninum* Liverpool]	−	+	−	+	−	−
64	CBZ49679.1	Elongation factor 1-alpha, related [*N. caninum* Liverpool]	−	+	+	+	−	−
65	CEL78445.1	ATP-dependent DNA helicase II, 70 kDa subunit, putative [*T. gondii* VEG]	−	+	−	−	−	−
66	CEL78039.1	TPA: pantothenate kinase, putative [*T. gondii* VEG]	−	−	+	−	−	−
67	CEL71084.1	Protein phosphatase 2C, putative [*N. caninum* Liverpool]	−	−	+	−	−	−
68	CBZ52637.1	Hypothetical protein NCLIV_024250 [*N. caninum* Liverpool]	−	−	+	−	−	−
69	CBZ53255.1	Rcn2-prov protein, related [*N. caninum* Liverpool]	−	−	−	−	+	−
70	CBZ53608.1	Heat shock protein 70, related [*N. caninum* Liverpool]	−	−	−	−	−	+

**+**, Protein identification as antigenic proteins; –, Protein identification as non-antigenic proteins; **+** ^**a**^, Protein identification as antigenic proteins specific with anti-*N. caninum* serum.

### Functional categorisation of immunoreactive proteins against *N. caninum*

To further understand the functions of the immunoreactive proteins against *N. caninum*, the 20 immunoreactive protein spots corresponding to 14 different specific antigens were putatively annotated using GO terms obtained from the ToxoDB Toxoplasma informatics resources (https://toxodb.org/toxo/app) and previous study reports (Table 4). The functional classification of the 14 different antigenic proteins against *N. caninum* is shown in Table 4. In the category of biological processes, eight proteins were associated with cell growth and invasion process, including HSP90-like protein, ubiquitin carboxyl-terminal hydrolase, microneme protein 4 (MIC4), inosine-5′-monophosphate dehydrogenase, actin, elongation factor 1-alpha, hypothetical protein NCLIV_005150 and peroxidoxin 2 (also called peroxiredoxin 2). In the molecular function category, four proteins corresponded to ATP, DNA or protein binding. Furthermore, a protein associated with cellular components and an unnamed protein product represented as rhoptry protein (ROP1) with no function data available were identified. Proteins involved in cell proliferation and invasion process were found to be immunogenic.

**Table 4:** Functional classification of immunoreactive proteins against *Neospora caninum.*

Accession No.	Protein name	Function	References
CBZ52122.1	HSP90-like protein, related [*N. caninum* Liverpool]	Plays an important role in growth and invasion	[42]
CBZ53664.1	Hypothetical protein NCLIV_034460 [*N. caninum* Liverpool]	ATP binding	ToxoDB
CEL75932.1	Ubiquitin carboxyl-terminal hydrolase, putative [*T. gondii* VEG]	Ubiquitin-dependent protein catabolic process	ToxoDB
CEL76857.1	corA-like Mg2 transporter domain-containing protein [*T. gondii* VEG]	Integral component of membrane	ToxoDB
CBZ49807.1	Putative microneme protein MIC4 [*N. caninum* Liverpool]	Important role in the early phase of host cell adhesion and invasion	[22]
CEL67492.1	Inosine-5′-monophosphate dehydrogenase [*N. caninum* Liverpool]	Oxidation-reduction process	ToxoDB
CBZ49859.1	Actin, related [*N. caninum* Liverpool]	Cellular processes	[3]
CBZ49956.1	Hypothetical protein NCLIV_004400 [*N. caninum* Liverpool]	Nucleic acid binding	ToxoDB
CBZ54957.1	Unnamed protein product [*N. caninum* Liverpool]	Rhoptry protein ROP1, unknown	ToxoDB
CBZ49679.1	Elongation factor 1-alpha, related [*N. caninum* Liverpool]	Plays an essential role in mediating host cell invasion	[45]
CBZ50039.1	Hypothetical protein NCLIV_005150 [*N. caninum* Liverpool]	Microtubule-based process	ToxoDB
CBZ52818.1	Armadillo/beta-catenin-like repeat-containing protein [*N. caninum* Liverpool]	Protein binding	ToxoDB
CBZ54937.1	Putative peroxidoxin 2 [*N. caninum* Liverpool]	Cell redox homeostasis, oxidation-reduction process	ToxoDB
CBZ52736.1	Putative Gbp1p protein [*N. caninum* Liverpool]	RNA binding	ToxoDB

## Discussion

*Toxoplasma*, *Cryptosporidium*, *Babesia* and *Neospora* are important veterinary pathogens that cause diseases in farm animals, resulting in considerable economic losses to the livestock sector [32]. *Toxoplasma gondii* is the most significant pathogen associated with reproductive problems, especially in small ruminants [14]; *C. parvum* is one of the most important causes of calf diarrhoea, particularly in neonatal calves [43]; and *Babesia* spp. cause tick-borne disease with a worldwide economic impact due to severe disease in cattle, among which *B. bovis* and *B. bigemina* are the two most important species [7]. Bovine neosporosis is a major cause of abortion in cattle worldwide, which causes serious economic losses to beef and dairy industries [15, 37]. Considering the lack of an effective treatment method or vaccine against neosporosis, there is a need to improve serodiagnostic methods to discriminate *N. caninum*-infected animals from those infected with other closely related pathogens in the assessment of epidemiology, surveillance and disease management [19].

The proteomics approach can help in the discovery of novel immunogens involved in host immune stimulation and can help in the identification of possible targets for drugs and vaccines [35]. High-resolution 2-DE protein separation combined with immunoblot analysis of antigenic proteins, followed by identification with MS and bioinformatics analysis provides an approach to identify parasite-specific proteins or distinct antigens that represent potential vaccine candidates or targets for serodiagnosis improvement [23, 25]. Although combinations of 2-DE, immunoblotting, and mass spectrometric analysis for analysing *N. caninum* antigens have been applied, there are limited studies on the identification of *N. caninum* antigenic proteins in the bovine host, a major victim of neosporosis. Lee *et al.* detected 102 antigen spots using IPG strips (pH 4–7) on immunoblot profile using serum from rabbit immunised with *N. caninum*, among which 17 spots corresponding to 11 antigenic proteins were identified as antigens from *N. caninum* on the 2-DE map [27]. Subsequently, 132, 84, 4 and 40 antigenic protein spots were recognised against bovine IgM, IgE, IgA and IgG, respectively, by immunoproteomics using serum from cow immunised with *N. caninum* [39]. Comparison of the antigenic proteome between *N. caninum* KBA-2 and VMDL-1 isolates using serum from cow immunised with *N. caninum* showed a high similarity pattern on 2-DE separation, and the antigenic spots on immunoblot profiles were also detected at similar locations in terms of p*I* and molecular weight [38]. In this study, we identified and analysed *N. caninum* (Nc-1 isolate) tachyzoite antigenic proteins recognised by each of apicomplexan-infected bovine sera, including *N. caninum*, *T. gondii*, *C. parvum*, *B. bovis* and *B. bigemina*, and healthy host sera on 2-DE immunoblot profiles. Based on 2-DE immunoblotting, we identified 20 antigenic spots corresponding to 14 specific antigens against *N. caninum*. Among these, HSP90, hypothetical protein NCLIV_034460, ubiquitin carboxyl-terminal hydrolase, corA-like Mg2 transporter domain-containing protein, microneme 4 (MIC4), inosine-5′-monophosphate dehydrogenase, actin, hypothetical protein NCLIV_004400, rhoptry protein (ROP1), elongation factor 1-alpha, hypothetical protein NCLIV_005150, armadillo/beta-catenin-like repeat-containing protein, peroxidoxin 2 and Gbp1p protein were significantly specifically immunoreactive corresponding to their immunoglobulin reactions against *N. caninum*.

Most antigenic proteins identified in this study were associated with cell invasion and proliferation processes of the parasite. Among these proteins, HSP90 is a molecule playing a vital role in the biology and virulence of the parasite. In *T. gondii*, HSP90 plays an important role in bradyzoite differentiation, host cell invasion, growth and virulence [42], whereas in *Plasmodium*, HSP90 was indicated as a protein regulating parasite growth in human erythrocytes [46]. Another previous report on HSP90 described species-specific antigens against *N. caninum* using sera from mice immunised with either *N. caninum* or *T. gondii* [48]. Elongation factor 1-alpha (EF-1α) is a key element of protein translation and one of the most abundant proteins expressed in eukaryotic cells [24]. Mice vaccinated with recombinant *T. gondii* EF-1α showed high levels of specific anti-*T. gondii* antibodies and production of IFN-gamma and interleukin-4, which significantly prolonged the survival time after challenge infection with the *T. gondii* RH virulent strain [45]. Although our results showed that EF-1α exhibits high immunoreactivity against *N. caninum*, we also found a strong reactivity pattern with anti-*T. gondii* on the 2-DE immunoblot profiles. NcMIC4 has been found to be largely upregulated in the *N. caninum* tachyzoite stage when entering and developing within the host cell, and re-expression of NcMIC4 occurred 30 min after entry into the host cell [22]. MIC1 and MIC4 induce protective immunity against *T. gondii* by stimulating the production of IL-2, IL-12, IFN-g and IL-10 in immunised mice, indicating that these proteins might become targets for the further development of vaccines [29]. Our study also showed that MIC4 reacted with *N. caninum*-infected bovine sera, indicating that it may be a promising candidate for diagnosis and vaccine development. However, it is necessary to evaluate the diagnostic and vaccine potential against bovine neosporosis in the near future.

Another immunoreactive protein identified in this study was actin, the protein responsible for several biological processes in apicomplexan parasites, including cell motility, host cell invasion, vesicular transport and apicoplast inheritance [11]. Actin strongly reacted with bovine IgM, IgG and IgE and exhibited immunodominant antigens with bovine IgG on the immunoblot profiles of both *N. caninum* KBA-2 and VMDL-1 isolates [38, 39]. In addition, it has been reported that there is *N. caninum* actin in at least nine different isoforms that are functional in cellular processes and might be regulated by mechanisms involving post-translational modifications [3]. Similarly, the armadillo/beta-catenin-like repeat-containing protein has been demonstrated to be crucial in apical rhoptry positioning and consequently aids in host cell invasion in *P. falciparum* and *T. gondii* [9, 31]. Ubiquitin plays an important role in protein turnover, cellular signalling and intracellular transport. It is conjugated to the lysine residues of proteins to regulate a large number of cellular processes [40]. A study on the ubiquitylation pathway in apicomplexan parasites suggested that ubiquitin is essential for controlling cellular processes throughout the apicomplexan complex parasitic life cycle [34]. Moreover, peroxidoxins and inosine-5′-monophosphate dehydrogenase exhibited high antigenic activity in our study and in other organisms as well [5, 10, 17], indicating their potential as vaccine candidates and drug targets. Interestingly, we discovered the unnamed protein product ROP1 exhibiting strong immunoreactivity against *N. caninum* but with no functional data available. Rhoptry proteins of apicomplexan pathogens play a vital role in parasite virulence. ROP5 was found to be critical for the pathogenesis of *T. gondii* in mice, as deletion of this gene attenuated virulence in the mice [36]. A recent report of *N. caninum* ROP5 knockout in a plaque assay indicated that *N. caninum* showed weakened invasion ability and slower intracellular growth, along with loss of virulence, in mice [30]. Similarly, *N. caninum* ROP2 was identified to play an essential role during host cell invasion processes and exhibits immunoprotective properties that induced host immune responses, indicating its potential as a vaccine candidate [12, 13]. Further study is required to clarify the function of ROP1 protein.

Although several studies have revealed the cross-reaction between *N. caninum* and *T. gondii*, there are limited studies describing cross-reactivity using proteomics among apicomplexan parasites in the bovine host. Three proteins, including PDI, HSP70 and RP1, were identified as cross-reactive antigens between *N. caninum* and *T. gondii* [28]. Some proteins showed high homology between *N. caninum* and *T. gondii* tachyzoites, such as HSP70, tubulin α- and β-chain, PDI, actin and enolase, which were believed to be conserved antigens in both parasites [26]. Zhang *et al.* also demonstrated that at least 18 protein spots showed cross-reaction between *N. caninum* and *T. gondii* using sera from mouse immunised with parasites and further found that some antigens shared high homology with the corresponding antigens of *T. gondii* [48]. In this study, a large number of *N. caninum* immunoproteomics profiles cross-reacted with *T. gondii* and *C. parvum* (Table 3). In addition, the corA-like Mg2 transporter domain-containing protein, elongation factor 1-alpha and ROP1 were found to be highly specific antigenic proteins against *N. caninum*, but these antigens were found in different spots recognised by other protozoan-infected sera, which had the same protein accession number. This finding might be attributed to the different forms of post-translational modification or different isoforms of these three proteins. In this study, as we used pooled bovine healthy sera, two protein spots reacted with the healthy sera, which might be due to non-specific binding of the bovine background antibody. Therefore, we deduced these two proteins as cross-reactive antigens. A high degree of cross-reactivity was identified in the antigens among these parasites, especially *N. caninum*, *T. gondii* and *C. parvum*.

Although many parasite antigens were identified in this study, there were several limitations to using this method. Since strong detergent was not added to the 2-DE gel electrophoresis buffer system, very high hydrophobic proteins were not able to dissolve and be detected. It was not possible to separate the proteins with very high or low isoelectric points by 2-DE gel electrophoresis. In addition, protein identification relied on protein visualisation by silver staining; therefore, very low-abundant proteins could not be found. As a result of these obstacles, other cross-reactive proteins might not be resolved and identified using this method.

## Conclusion

There is a need for specific biomarkers in veterinary medicine for diagnosis and follow-up treatment. Immunoproteomics is very useful for identifying host immune responses and characteristics of individual antigenic proteins. This study demonstrated the detection of disease-specific proteins using infected bovine sera, which exhibited distinct specific antigens against *N. caninum* and possible cross-reactive antigens with other apicomplexan parasites, especially *T. gondii* and *C. parvum*. Further study is required to evaluate cross-reactive antigens as potential common vaccine candidates or drug targets to control the diseases caused by these parasites in the bovine host. Therefore, we can target these highly specific immunoreactive antigens for further identification and characterisation in immunodiagnosis and vaccine development.
